# The structure of AcrIC9 revealing the putative inhibitory mechanism of AcrIC9 against the type IC CRISPR–Cas system

**DOI:** 10.1107/S2052252523007236

**Published:** 2023-09-01

**Authors:** Yong Jun Kang, Ju Hyeong Kim, Gwan Hee Lee, Hyun Ji Ha, Young-Hoon Park, Eunmi Hong, Hyun Ho Park

**Affiliations:** a College of Pharmacy, Chung-Ang University, Seoul 06974, Republic of Korea; b Department of Global Innovative Drugs, Graduate School of Chung-Ang University, Seoul 06974, Republic of Korea; c New Drug Development Center, Daegu-Gyeongbuk Medical Innovation Foundation, Daegu 41061, Republic of Korea; Chinese Academy of Sciences, China

**Keywords:** anti-CRISPR, AcrIC9, bacterial immunity, CRISPR–Cas systems, type IC cascades, inhibitory mechanisms, DNA-mimic Acrs

## Abstract

CRISPR–Cas systems are known to be part of the bacterial adaptive immune system that provides resistance against intruders such as viruses, phages and other mobile genetic elements. To combat this bacterial defense mechanism, phages encode inhibitors called Acrs (anti-CRISPR proteins) that can suppress them. AcrIC9 is the most recently identified member of the AcrIC family that inhibits the type IC CRISPR–Cas system. Here, the crystal structure of AcrIC9 from *Rhodobacter capsulatus* is reported, which comprises a novel fold made of three central antiparallel β-strands surrounded by three α-helixes, a structure that has not been detected before. It is also shown that AcrIC9 can form a dimer via di­sulfide bonds generated by the Cys69 residue. Finally, it is revealed that AcrIC9 directly binds to the type IC cascade. Analysis and comparison of its structure with structural homologs indicate that AcrIC9 belongs to DNA-mimic Acrs that directly bind to the cascade complex and hinder the target DNA from binding to the cascade.

## Abstract 2

The high-resolution crystal structure of AcrIC9 is elucidated and its inhibitory mechanism on the type IC CRISPR–Cas system is unveiled. Analysis and comparison of its structure with structural homologs indicate that AcrIC9 belongs to DNA-mimic Acrs (anti-CRISPR proteins) that directly bind to the cascade complex and hinder the target DNA from binding to the cascade.

## 1. Introduction

As a result of the long war between bacteria and its invaders, such as viruses and mobile genetic elements, bacteria have armed themselves with CRISPR–Cas systems that can remove genetic material introduced into the host cell via infection (Horvath & Barrangou, 2010 ▸; Brouns *et al.*, 2008 ▸; Hampton *et al.*, 2020 ▸). The CRISPR genetic material is composed of host-encoded short repeats separated by unique sequences that are derived from previous viral infections. It is used as genetic information for detecting similar genetic material and destroying the infectious agent in subsequent infections (Sorek *et al.*, 2008 ▸; Barrangou *et al.*, 2007 ▸; Mojica & Rodriguez-Valera, 2016 ▸). Due to their mechanistic similarity, the bacterial CRISPR–Cas system is considered to be the bacterial equivalent of the adaptive immune system of higher organisms, which records memories of past infections and executes a fast immune response to subsequent ones (Jackson *et al.*, 2017 ▸).

The diverse CRISPR–Cas systems found in various bacteria and archaea have been categorized into two classes according to the organization of the CRISPR locus and Cas genes (Makarova *et al.*, 2020 ▸). The biggest difference between the class 1 and class 2 systems is that multi-subunit Cas protein complexes are used to perform multiple functions in the former while a single multi-domain Cas protein is used to perform multiple activities in the latter (Makarova *et al.*, 2020 ▸). These two classes can be further grouped into six types (I to VI) according to their action mechanism and phylogeny. Types I, III and IV are in the class 1 group while types II, V and VI are in the class 2 group (Makarova *et al.*, 2020 ▸). The CRISPR–Cas type I systems have been studied the most because they are the most abundant and the most widely distributed among various bacterial species. They have been further divided into seven subtypes (A to G) based on the organization of the signature Cas proteins and their composition (Makarova *et al.*, 2020 ▸). The specific features of the type I systems are a multiple Cas protein complex called a CRISPR-associated complex for antiviral defense (cascade) and a small RNA fragment [called CRISPR (cr)RNA] encoded by the bacterial CRISPR sequence. When the cascade is formed, it recognizes an invader’s DNA through the crRNA and collects *trans*-acting nuclease Cas3 to cleave the target DNA (Brouns *et al.*, 2008 ▸). Due to the ability of the CRISPR–Cas system to cleave the specific DNA sequence based on the sequence information provided by the crRNA therein, this specific DNA-cutting mechanism has been applied to gene editing in higher organisms and is considered to be a promising tool for treating genetic diseases (Hsu *et al.*, 2014 ▸; Wang *et al.*, 2013 ▸; Knott & Doudna, 2018 ▸).

Anti-CRISPR proteins (Acrs) are naturally occurring inhibitors of CRISPR–Cas systems that have been identified in various bacterial and archaeal species (Malone *et al.*, 2021 ▸; Wiegand *et al.*, 2020 ▸). Their discovery and characterization have provided a powerful tool for controlling the activity of CRISPR–Cas systems, thereby improving the safety and precision of genome editing. Since the first Acr was reported in phages in 2013 (Bondy-Denomy *et al.*, 2013 ▸), more than 100 have been identified through functional screening and bio­informatic tools (Borges *et al.*, 2017 ▸; Eitzinger *et al.*, 2020 ▸; Bondy-Denomy *et al.*, 2018 ▸). Because Acrs do not share high sequence homology or contain any common structural motifs that can be used for protein classification, they have been classified solely based on their targeted CRISPR–Cas system (Borges *et al.*, 2017 ▸; Wiegand *et al.*, 2020 ▸). Based on this strategy, Acrs that inhibit the type IC CRISPR–Cas system have been classified as AcrIC. For instance, AcrIC9 from *Rhodobacter capsulatus* is the most recently identified AcrIC family member that inhibits the type IC CRISPR–Cas system (Gussow *et al.*, 2020 ▸). The expression of many *acr* genes is controlled by putative transcriptional regulators called anti-CRISPR-associated proteins (Acas) (Birkholz *et al.*, 2019 ▸; Stanley *et al.*, 2019 ▸), which are thus indirect regulators of CRISPR–Cas systems (Lee *et al.*, 2022 ▸; Malone *et al.*, 2021 ▸). Because Acrs and Acas regulate the activity of CRISPR–Cas systems, they are considered to be promising tools for controlling gene editing using CRISPR–Cas (Marino *et al.*, 2020 ▸).

Although many different inhibitory mechanisms of the Acrs on the CRISPR–Cas systems have already been revealed (Wiegand *et al.*, 2020 ▸; Zhu *et al.*, 2018 ▸; He *et al.*, 2018 ▸; Chowdhury *et al.*, 2017 ▸; Kim *et al.*, 2022 ▸), that of the AcrIC family is not yet fully understood due to the lack of structural information. The only available information on this family is the most recently revealed structure of AcrIC5 (Kang & Park, 2022 ▸). In this study, we determined the crystal structure of AcrIC9 from *R. capsulatus*, which is the second member of the AcrIC family to have its structure elucidated. The unique structural features of AcrIC9 and its comparison with structural homologs helped to tentatively reveal the working mechanism of its inhibitory mode of action on the type IC CRISPR–Cas system. Uncovering the structure of AcrIC9 and its mode of interaction in the type IC cascade will help to elucidate the diversity of the inhibitory mechanisms of the Acr family and enable the fine-tuning of gene-editing-based therapeutic applications.

## 2. Materials and methods

### 2.1. Cloning, protein expression and purification

The *acrIC9* gene encoded by residues 1–79 in *R. capsulatus* (GenBank ID ETD02882) was synthesized by Bionics (Daejeon, Republic of Korea). The synthesized gene was cloned into a pET21a plasmid vector (Novagen, Madison, WI, USA) by using the NdeI and XhoI restriction sites. The resulting recombinant AcrIC9-expressing construct was introduced into *Escherichia coli* BL21(DE3) competent cells for transformation. The transformed cells were then grown on a lysogeny broth (LB) agar plate containing 100 µg ml^−1^ of ampicillin for 18 h at 37°C in an incubator. A single bacterial colony was picked using a sterilized pipet tip and suspended in 5 ml of LB medium containing 100 µg ml^−1^ of ampicillin. A 100 µl aliquot of the grown bacterial cells was transferred to 1 l of LB medium for further culturing until the optical density at 600 nm (OD600) reached ∼0.7. Next, 0.25 m*M* IPTG used for inducing the expression of the *acrIC9* gene was added. IPTG-treated cells were then grown for 18 h at 20°C in a shaking incubator.

AcrIC9-overexpressing cells were harvested via centrifugation at 2000*g* for 15 min at 20°C, resuspended in 20 ml buffer A (20 m*M* Tris–HCl pH 8.0 and 500 m*M* NaCl) and lysed via ultrasonication. The lysed cells were removed via centrifugation at 10 000*g* for 30 min at 4°C. Afterward, the supernatant was collected and mixed with a nitrilo­tri­acetic acid (NTA)-affinity resin (Qiagen, Hilden, Germany) for 2 h. The mixture was then poured into a gravity-flow column (Bio-Rad, Hercules, CA, USA). The target protein-bound Ni-NTA resin in the column was washed with 40 ml of buffer A to remove unbound protein. Finally, 0.6 ml × 5 buffer B (20 m*M* Tris–HCl pH 8.0, 500 m*M* NaCl and 250 m*M* imidazole) was added to the column to elute the target protein from the resin.

Purified AcrIC9 was concentrated to 18 mg ml^−1^ and loaded onto a Superdex 200 10/300 GL column (GE Healthcare, Waukesha, WI, USA) in an ÄKTA Explorer system (GE Healthcare) to conduct size-exclusion chromatography (SEC). The system had been pre-equilibrated with SEC buffer (20 m*M* Tris–HCl pH 8.0 and 150 m*M* NaCl). The proper peak fractions from SEC containing the AcrIC9 target protein were picked, pooled and then concentrated to 7.0 mg ml^−1^ for further experimentation. SDS–PAGE was used to analyze the purity of the protein sample.

### 2.2. Multi-angle light scattering (MALS) analysis

The accurate molecular weight of AcrIC9 in solution was measured using SEC–MALS. The purified AcrIC9 protein in SEC buffer was loaded onto a Superdex 200 Increase 10/300 GL SEC column (GE Healthcare) connected on the ÄKTA Explorer system (GE Healthcare) connected to a DAWN-TREOS MALS detector (Wyatt Technology, Santa Barbara, CA, USA). MALS experiments were performed at room temperature. The molecular weight of bovine serum albumin was used as a reference. The obtained low MALS data were processed using *ASTRA* software provided by the manufacturer.

### 2.3. Crystallization and data collection

The hanging-drop vapor diffusion method was employed to crystallize the purified AcrIC9 protein. Crystallization was performed at 20°C and crystal plates were incubated at 20°C. X-ray diffractable crystals were obtained by equilibrating a mixture containing 1 µl of protein solution (7.0 mg ml^−1^ of protein in SEC buffer) and 1 µl of reservoir solution containing 2 *M* ammonium sulfate, 0.1 *M* cacodylate, and 0.2 *M* sodium chloride (pH 6.5) against 500 µl of reservoir solution. The crystals appeared within a day. X-ray diffraction data were collected at the beamline BL-5C in the Pohang Accelerator Laboratory (Pohang, Korea). *HKL2000* software was used for data processing (Otwinowski, 1990 ▸).

### 2.4. Structure determination and analysis

The *Phaser* program in the *PHENIX* package (Adams *et al.*, 2011 ▸) was used to determine the structure of AcrIC9 by using the molecular replacement (MR) phasing method (McCoy, 2007 ▸). The search model for MR was generated via *AlphaFold*2 (Jumper *et al.*, 2021 ▸). The initial model was built automatically using *AutoBuild* from the *PHENIX* package (Terwilliger *et al.*, 2008 ▸), and further model building with refinement was performed using *Coot* (Emsley & Cowtan, 2004 ▸) and *phenix.refine* (Afonine *et al.*, 2012 ▸). The quality of the structure and stereochemistry of the model were analyzed by using *MolProbity* (Williams *et al.*, 2018 ▸). *PyMOL* was used to generate the structural figures (DeLano & Lam, 2005 ▸).

### 2.5. Mutagenesis

A quick-change kit (Stratagene) was used for site-directed mutagenesis. The manufacturer’s protocol was used to introduce mutation in AcrIC9. The mutants were confirmed via sequencing by Bionics (Seoul, Republic of Korea). Each mutant protein was prepared using the same method employed for purifying the wild-type protein.

### 2.6. Surface plasmon resonance (SPR) spectroscopy

This was conducted using a BIAcore T200 with an NTA sensor chip and HBS-P buffer [0.01 *M* HEPES pH 7.4, 0.15 *M* NaCl and 0.005%(*v*/*v*) Surfactant P20] for the sample and running buffer (Cytiva). The analysis temperature and sample compartment were set to 25°C. The NTA surfaces were washed with 0.35 *M* EDTA prior to loading with 0.5 m*M* NiCl_2_. For direct NTA chip capture, following 1 min injection of 0.5 m*M* NiCl_2_, the purified NlaCascade/crRNA complex at a concentration of 50 µg ml^−1^ diluted in HBS-P buffer was injected with a flow rate of 10 µl min^−1^ to achieve a capture level of 711–826 resonance units. To study interactions in the NlaCascade/crRNA complex, AcrlC9 was diluted twofold from 125 n*M* and injected over the surfaces at 30 µl min^−1^ for a contact time of 120 s, followed by dissociation for 240 s. The running sensorgrams were blank subtracted prior to fitting to a 1:1 binding model. The association rate constant (ka), dissociation rate constant (kd) and equilibrium dissociation constant (KD = kd/ka) were determined based on the results.

### 2.7. Structural data accessibility

Coordinate and structural factors were deposited in the Protein Data Bank under PDB ID 8hjj.

## 3. Results

### 3.1. Purification and biochemical characterization of AcrIC9

Because of its narrow distribution and late discovery, the type IC CRISPR–Cas system has been the least studied of the CRISPR–Cas type I systems. Moreover, the structures of the AcrIC family members that inhibit the type IC CRISPR–Cas system have not yet been determined, and so the inhibitory mechanism was not understood at the molecular level until now.

**Figure 1:**
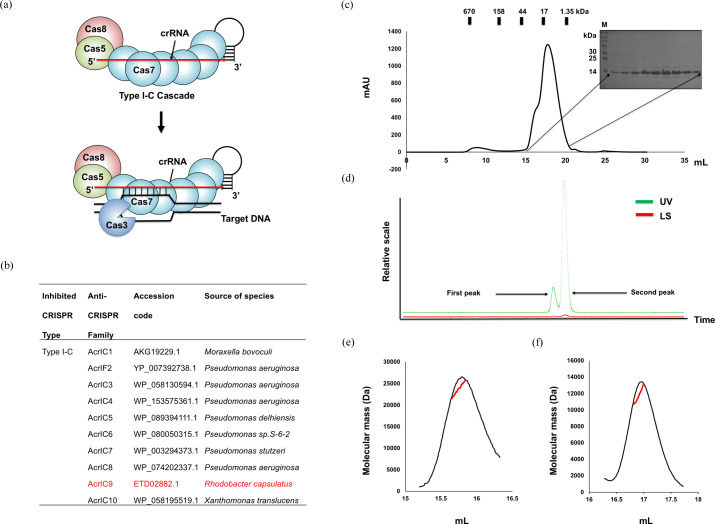
Purification and characterization of AcrIC9 in solution. (*a*) Schematic diagrams of the type IC CRISPR–Cas system and the target for AcrIC9. The crRNA that acts as a scaffold for the type IC cascade is shown by the red line. (*b*) A table summarizing the ten AcrIC families identified so far. The AcrIC9 characterized in this study is in red. (*c*) A SEC profile of AcrIC9. The inset is a photograph of an SDS–PAGE gel loaded with extracts from the SEC peak fractions. The corresponding SEC fractions are indicated by the black arrows. M indicates the protein size marker. (*d*) A graphical representation of the data obtained by using SEC–MALS (LS, light scattering). Below this there is experimental analysis of the results obtained from the MALS analysis of (*e*) the first peak and (*f*) the second peak. The experimental MALS data (red line) are plotted as the SEC elution volume (*x* axis) versus the absolute molecular mass (*y* axis) distribution on the SEC chromatogram (black) at 280 nm.

The main feature of the type IC CRISPR–Cas system is a minimal cascade complex composed of Cas5, Cas7, Cas8 and crRNA (Csörgő *et al.*, 2020 ▸). However, it does not contain Cas6, which is an RNase responsible for processing crRNA and has usually been detected in the cascades of other CRISPR–Cas type I subtypes [Fig. 1 ▸(*a*)]. So far, ten AcrIC families that have the potential to inhibit the type IC CRISPR–Cas system have been discovered [Fig. 1 ▸(*b*)] (León *et al.*, 2021 ▸; Gussow *et al.*, 2020 ▸). To understand the molecular mechanism of type IC CRISPR–Cas inhibition by the AcrIC family via a structural study, recently identified AcrIC9 from *R. capsulatus* was purified by using a quick two-step chromatography system comprising affinity chromatography followed by SEC. Since AcrIC9 with a molecular weight of 11.21 kDa eluted in between myoglobin (∼17 kDa) and vitamin B12 (∼1.350 kDa) during SEC, we speculated that AcrIC9 exists as a monomer in solution [Fig. 1 ▸(*c*)]. A shoulder in the SEC profile was observed in front of the main peak at ∼16 ml, which could signify a dimer. Because determining the functional stoichiometry of Acrs is critical for understanding their molecular mechanism, we analyzed it by employing MALS, which can measure the absolute molecular mass of a protein particle in solution. During the SEC–MALS process, a small peak was detected just before the main peak in the SEC profile [Fig. 1 ▸(*d*)]; we determined that their absolute molecular weights were 23.7 kDa (with a fitting error of 7.8%) with a polydispersity value of 1.004 [Figs. 1 ▸(*e*)] and 11.7 kDa (with a fitting error of 8.9%) with a polydispersity value of 1.000 [Fig. 1 ▸(*f*)], respectively. These results indicate that the first and second peaks are the dimeric and monomeric forms of AcrIC9, respectively. Judging by the heights of the peaks, the major form of AcrIC9 is the monomer. However, the small amount of dimer should not be neglected because it has been reported that the dimeric forms of several Acr family members are critical for their activity (He *et al.*, 2018 ▸; Pawluk *et al.*, 2017 ▸; Wang *et al.*, 2016 ▸).

### 3.2. The overall structure of AcrIC9

**Figure 2:**
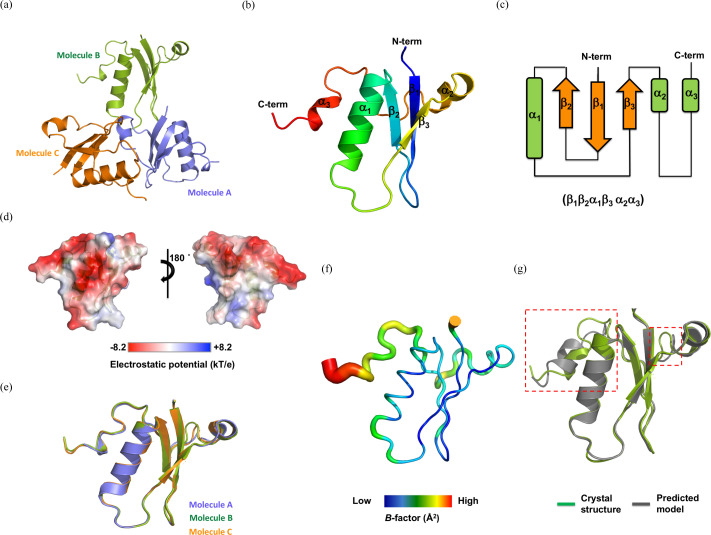
Schematic diagrams of the structure of AcrIC9 from *R. capsulatus*. (*a*) The AcrIC9 structure showing the three molecules detected in the crystallographic unit. (*b*) The structure of the AcrIC9 monomer. The color of the structure from the N- to the C-termini gradually changes from blue to red through the spectrum. (*c*) A topological representation of the AcrIC9 structure. The order of the secondary structure of α-helixes and β-sheets is shown in parentheses. (*d*) The surface electrostatic potential of AcrIC9 showing the surface electrostatic distribution: −8.2 *kT* e^−1^ (red) to 8.2 *kT* e^−1^ (blue). (*e*) Superimposition of the three molecules in the same crystallographic unit cell. (*f*) *B*-factor distribution for the AcrIC9 structure. The structure is shown as a putty representation. (*g*) Structural comparison of the crystal structure (green) with the predicted structure (gray) generated as a cartoon by using *AlphaFold*2. The main parts of the backbones that are not perfectly aligned are indicated by red-dashed boxes.

AcrIC9 was successfully crystalized as a single crystal diffracting at 2.4 Å under synchrotron radiation. The final structural model of AcrIC9 was refined to *R*_work_ = 21.44% and *R*_free_ = 26.92%. Details of the diffraction data and refinement statistics are summarized in Table 1 ▸. The crystal belongs to space group *P*3_1_12, with three molecules present in the asymmetric unit (ASU). The final structural models contain residues Met5 to Ser76 for molecule A and Met5 to Glu78 for molecules B and C [Fig. 2 ▸(*a*)]. The overall structure of AcrIC9 comprises three antiparallel β-sheets (β1–β3) and three α-helixes (α1–α3) [Figs. 2 ▸(*b*) and 2 ▸(*c*)]. The results of a topological analysis indicate that the fold of AcrIC9 is formed by three antiparallel β-sheets surrounded by three α-helixes in the order β1β2α1β3α2α3, which implies a unique fold [Fig. 2 ▸(*c*)]

Since the electrostatic surface features of a protein are sometimes correlated with its function, we analyzed them to obtain information about the working mechanism of AcrIC9. The results of this analysis indicate that the surface of AcrIC9 is highly acidic [Fig. 2 ▸(*d*)]. A small and highly acidic surface has been detected on many Acr family members, which enables inhibition of the CRISPR–Cas system by directly binding to a specific target DNA-binding site, thereby mimicking the DNA property (Liu *et al.*, 2019 ▸; Rollins *et al.*, 2019 ▸). Therefore, this highly acidic surface feature may indicate that AcrIC9 is one of the DNA-mimic Acrs.

The structures of all three molecules in the same ASU were very similar to each other, with root-mean-squared deviation (RMSD) values of 0.35 Å between molecules A and B and 0.32 Å between molecules A and C [Fig. 1 ▸(*e*)]. Moreover, the long α2–α3 connecting loop and C-terminal loop exhibit a relatively high *B*-factor value (average of 54.3 Å^2^) compared with the low *B* factor (average of 40.2 Å^2^) of the overall structure. This indicates that although a major part of AcrIC9 is rigid in solution, it contains a relatively flexible α2–α3 connecting loop [Fig. 2 ▸(*f*)].

Because the structure of AcrIC9 was solved via MR using the predicted structural model generated by using *AlphaFold*2 as the search model, we were curious about how similar these structures were. To answer this question, we compared them via structural superposition. As shown in Fig. 2 ▸(*g*), although most of the main backbone of the predicted structure was identical to that of the crystal structure, the C-terminal part including the long α2–α3 connecting loop and α3 in the latter did not superpose well onto that of the former, indicating that the main backbone of the C-terminal part is flexible and hard to be predicted for one conformation due to its flexible nature.

### 3.3. AcrIC9 protein forms dimer in solution via di­sulfide bond

**Figure 3:**
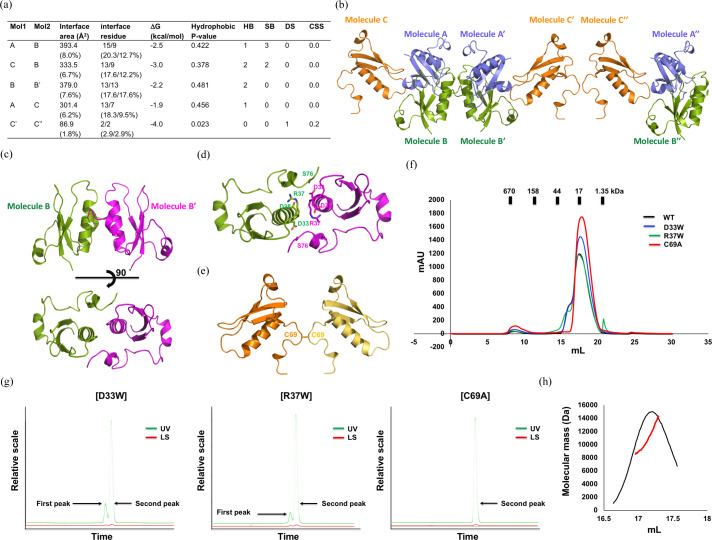
The tentative structure of the AcrIC9 dimer in solution. (*a*) A table summarizing the results using the *PISA* server. (*b*) Crystallographic packing symmetry via *PyMOL* showing the three AcrIC9 molecules (A, B and C) found in the ASU. Their other symmetries are labeled with ′ or ′′. (*c*) A schematic diagram of the putative dimeric structure of the B/B′ complex. (*d*) Details of the interactions in the B/B′ complex. (*e*) A schematic diagram of the putative dimeric structure of the C′/C′′ complex (Cys69: the residue involved in di­sulfide bond formation). (*f*) SEC profiles of wild-type AcrIC9 and various mutations of the putative interface binding sites. (*g*) A graphical representation of the SEC–MALS data for the putative interface binding sites of the various mutants (D33W, R37W and C69A). The first and second peaks generated for the dimer and monomer of AcrIC9 are indicated by the black arrows. (*h*) Experimental analysis of the MALS results for the SEC peaks generated from the C69A mutant. The experimental MALS data (red line) are plotted as the SEC elution volume (*x* axis) versus the absolute molecular mass (*y* axis) distribution on the SEC chromatogram (black) at 280 nm.

Although the majority of Acrs [including the most recently reported AcrIC5 (Kang & Park, 2022 ▸)] inhibit CRISPR–Cas systems in their monomeric forms, it has also been shown that the dimeric form of certain Acrs is critical for their functionality (He *et al.*, 2018 ▸; Pawluk *et al.*, 2017 ▸; Wang *et al.*, 2016 ▸). Although most of the AcrIC9 in solution was in the monomer form, its SEC–MALS profile shows a tentative peak indicating the dimer form [Figs. 1 ▸(*d*) and 1 ▸(*e*)]. Thus, we analyzed its potential protein–protein interactions (PPIs) using the *PDBePISA* PPI-calculating server (Krissinel & Henrick, 2007 ▸) and used the results to suggest several tentative dimer structures [Fig. 3 ▸(*a*)]. Accordingly, the interface formed by molecules C′ and C′′ achieved a complex similarity score (CSS) of 0.2 (scores range from 0 to 1 as the interface relevance to the complex formation increases), which suggests that it is the most appropriate tentative dimer since the CSSs of the other possible PPIs were 0.0 [Fig. 3 ▸(*a*)]. These calculations imply that the PPI formed between molecules C′ and C′′ is the most significant interaction force for forming the tentative dimer of AcrIC9.

The structure of the tentative dimer was further investigated by analyzing the crystallographic packing to find the positions of molecules B′, C′ and C′′ [Fig. 3 ▸(*b*)]. The results indicate that the B/B′ and C′/C′′ dimers form a symmetric dimer with a twofold axis. Interestingly, the buried interface area of the C′/C′′ dimer was only 1.8%, which is too small for proper dimer formation, while that of the B/B′ dimer was 7.6% [Figs. 3 ▸(*a*) and 3 ▸(*c*), respectively], thereby indicating that it is the best candidate. To determine which dimeric form is the most realistic in solution, we analyzed the PPI of B/B′ in detail to obtain structural information. The main force maintaining the B/B′ dimer is the formation of hydrogen bonds between Asp33, Arg37 and Ser76 [Fig. 3 ▸(*d*)], while that maintaining the C′/C′′ dimer is due to the formation of di­sulfide bonds between the two cysteine groups [Fig. 3 ▸(*e*)]. Based on this structural analysis, we mutated three residues, R33W and R37W for disrupting the B/B′ dimer and C69A for disrupting the C′/C′′ dimer, and then performed SEC–MALS to determine which of them negatively affects dimer formation. We observed that the SEC profile of the C69A mutant did not contain the dimer peak whereas those of the D33W and R37W mutants did [Fig. 3 ▸(*f*)]. Moreover, the MALS analysis provided exactly the same result [Fig. 3 ▸(*g*)]. The absolute molecular weight from the main peak produced by C69A confirmed via MALS was 11.5 kDa (with a fitting error of 3.5%) [Fig. 3 ▸(*h*)], indicating that the dimer was disrupted by mutating the Cys69 residue to alanine, and thus the C′/C′′ dimer is the most likely form in solution.

### 3.4. AcrIC9 directly interacts with the type IC cascade from *Neisseria lactamica* via Cas7

**Figure 4:**
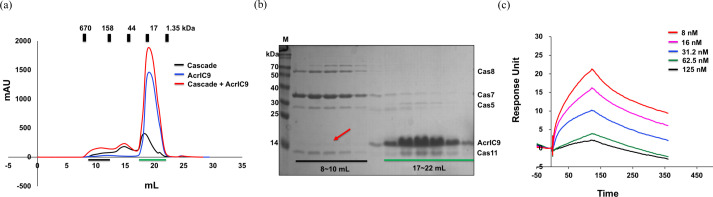
Direct interaction between AcrIC9 and the type IC cascade. (*a*) Interaction analysis between AcrIC9 and the type IC cascade by using SEC. The blue, black and red lines indicate AcrIC9, the cascade, and a mixture of AcrIC9 and the cascade, respectively. (*b*) SDS–PAGE gels produced after loading the SEC fractions of the two peaks (8–10 ml and 17–22 ml) from the mixed sample (the cascade + AcrIC9) shown in (*a*). The positions of each cascade subunit are indicated on the gel. AcrIC9 that co-migrated with the cascade is indicated by the red arrow. The corresponding fractions loaded onto the SDS–PAGE gels are indicated by the black and green bars. (*c*) Binding of AcrlC9 to the cascade complex captured on an NTA chip by using SPR. The cascade complex was captured on an NTA chip and AcrlC9 was bound to the cascade complex in the concentration range from 8 to 125 n*M*.

Due to the acidic nature of AcrIC9 and the fact that direct binding to the cascade is the most frequent inhibition mechanism by the Acr family (Rollins *et al.*, 2019 ▸; Liu *et al.*, 2019 ▸), we tested the direct interaction between AcrIC9 and the type IC cascade. Since we could not access the purified type IC cascade from *Pseudomonas aeruginosa*, in which the type IC cascade is reportedly suppressed by AcrIC9 (Gussow *et al.*, 2020 ▸), we used the one from *N. lactamica* instead for a direct *in vitro* binding assay. Although no clear peaks indicating formation of the complex were present in the SEC profile after mixing AcrIC9 and the type IC cascade [Fig. 4 ▸(*a*)], we found that the former co-eluted with the type IC cascade complex at ∼8–10 ml, as suggested by the SDS–PAGE results in Fig. 4 ▸(*b*). In the absence of the cascade, AcrIC9 did not elute in the 8–10 ml fraction (Fig. S1 of the supporting information), while, in the absence of AcrIC9, the cascade peak in the SEC profile did not contain the AcrIC9 protein (Fig. S2). We performed SPR spectroscopy to confirm the interaction between AcrIC9 and the cascade. As shown in Fig. 4 ▸(*c*) and Table S1 of the supporting information, AcrIC9 binds to the cascade complex with a KD value of 4.32 n*M*. These experimental data indicate that AcrIC9 directly binds to the type IC cascade, which might be related to the inhibitory function of AcrIC9.

**Figure 5:**
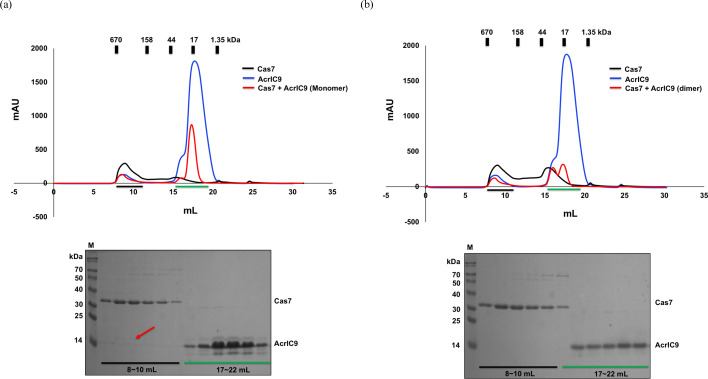
Evidence showing that AcrIC9 binds directly to Cas7. (*a*), (*b*) Interaction analysis between (*a*) monomeric or (*b*) dimeric AcrIC9 and cascade subunit Cas7 via SEC. The black, red and blue lines indicate Cas7, a mixture of AcrIC9 and Cas7, and AcrIC9, respectively. The SDS–PAGE gels produced for the mixture of AcrIC9 and Cas7 are provided under the SEC profiles. The loaded SEC fractions on the SDS–PAGE gels are indicated by black and green bars, while the co-migrated AcrIC9 band is indicated by the red arrow.

The next questions to answer were how AcrIC9 binds to the type IC cascade and which components it interacts with. To answer them, we once again performed SEC followed by SDS–PAGE with a mixture of purified components from the cascade (Cas5 and Cas7) and AcrIC9 to determine whether any co-migration patterns indicating complex formation were present. The results show that AcrIC9 co-eluted with Cas7 but not with Cas5 [Fig. 5 ▸(*a*) and Fig. S3]. In the absence of AcrIC9, the band corresponding to it was missing on the SDS–PAGE gel (Fig. S4). Interestingly, the dimer form of AcrIC9 did not co-elute with Cas7 [Fig. 5 ▸(*b*)], indicating that only the monomer form of AcrIC9 interacts with it in the type IC cascade.

### 3.5. The putative model for the inhibitory mode of action of AcrIC9 on the type IC cascade

**Figure 6:**
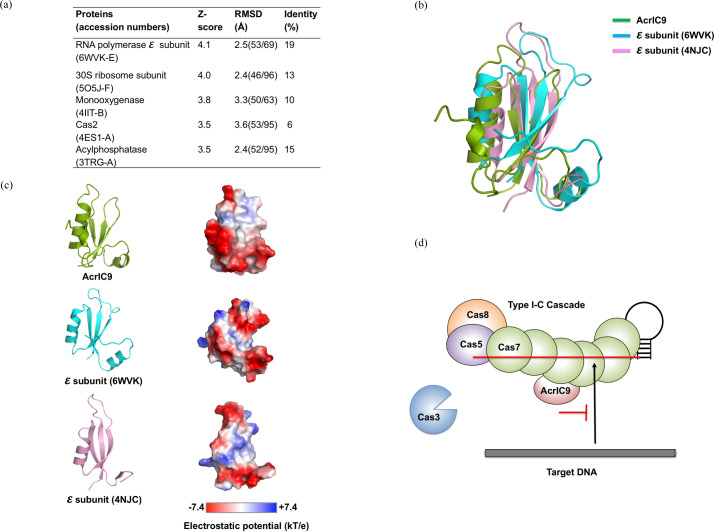
The proposed model for the inhibitory mode of action of AcrIC9 on the type IC CRISPR–Cas system. (*a*) A table summarizing the results of the *DALI* search. (*b*) Structural comparison of AcrIC9 with the ε subunits of RNA polymerase from *Bacillus subtilis* (PDB ID 6wvk) and *Geobacillus stearothermophilus* (PDB ID 4njc; Keller *et al.*, 2014 ▸) by superimposition. (*c*) A surface electrostatic potential comparison of AcrIC9 with the ε subunits of RNA polymerase from *B. subtilis* (PDB ID 6wvk) and *G. stearothermophilus* (PDB ID 4njc). A schematic representation of the structures of AcrIC9 and the two ε subunits is presented on the left side. Electrostatic potential mapping [−7.4 *kT* e^−1^ (red) to 7.4 *kT* e^−1^ (blue)] of the three different proteins is shown on the right. (*d*) The putative model for the inhibitory mode of action of AcrIC9 on the type IC CRISPR–Cas system.

To uncover clues that help to understand the inhibitory process of AcrIC9 on the type IC CRISPR–Cas system, we searched for structural homologs using the *DALI* server (Holm & Laakso, 2016 ▸). The top five matches were the ε subunit of RNA polymerase (PDB ID 6wvk-E; Newing *et al.*, 2020 ▸), the 30S ribosome subunit (PDB ID 5o5j-F; Hentschel *et al.*, 2017 ▸ ), monooxygenase (PDB ID 4iit-B; Grishin *et al.*, 2013 ▸), Cas2 (PDB ID 4es1; Nam *et al.*, 2012 ▸) and acyl­phosphatase (PDB ID 3trg; Franklin *et al.*, 2015 ▸) [Fig. 6 ▸(*a*)]. The search results based on low *Z* scores ranging from 4.1 to 3.5, high RMSD values ranging from 2.4 to 3.6 Å, and low sequence identities ranging from 6 to 19% indicate that the structural similarity between the top matches and AcrIC9 was fairly low, thereby indicating the novel structure of the latter.

Because the structure of the RNA polymerase ε subunit is the most structurally similar to AcrIC9, we compared the structure of the latter with two structures of the former via structural superposition. This comparison shows that their structures are similar in that all three have a structural fold composed of central antiparallel β-strands surrounded by two α-helixes [Fig. 6 ▸(*b*)]. The results from an electrostatic surface analysis also indicate that the acidic feature of the surface of AcrIC9 is similar to that of the ε subunit of RNA polymerase [Fig. 6 ▸(*c*)]. Because it has been reported that the ε subunit is one of the accessory components that controls the access of RNA polymerase to DNA (Newing *et al.*, 2020 ▸; Keller *et al.*, 2014 ▸), we speculate that AcrIC9 also inhibits DNA access by binding to the cascade.

## 4. Discussion

The structures and inhibitory mechanisms of many Acrs have been characterized (Wiegand *et al.*, 2020 ▸; Zhu *et al.*, 2018 ▸; He *et al.*, 2018 ▸; Chowdhury *et al.*, 2017 ▸) due to their importance as endogenous regulators of CRISPR–Cas-based gene-editing technology (Malone *et al.*, 2021 ▸; Wiegand *et al.*, 2020 ▸; Marino *et al.*, 2020 ▸; Gussow *et al.*, 2020 ▸). Although various inhibitory mechanisms of Acrs against the CRISPR–Cas systems have been elucidated, that of the AcrIC family members has not due to the lack of structural information. The only one with an elucidated structure at the moment is AcrIC5, which was published in the middle of 2022 by our group (Kang & Park, 2022 ▸). In the present study, we characterized and elucidated the structure of another member of the AcrIC family, AcrIC9. Structural analysis results show that AcrIC9 is composed of novel folds comprising three central antiparallel β-sheets surrounded by three α-helixes, while the results of an electrostatic surface analysis indicate that AcrIC9 has a highly acidic surface. Although AcrIC9 in solution is mostly in the monomeric form, we elucidated that AcrIC9 can form a dimer mediated by di­sulfide bonds. However, given that the intracellular space is characterized by a reducing environment, it is unlikely that the dimer is formed.

To confirm whether the di­sulfide bond-mediated dimerization of AcrIC9 is a general mechanism for the AcrIC9 family, we analyzed the cysteine residue to ascertain whether it is conserved in the homologs. The results indicated that this aspect of AcrIC9 is not conserved in different species. Two proteins with the most similarity were identified as uncharacterized ones in *Phormidium sp.* and *Agrobacterium tumefaciens*. Although the sequence identity between AcrIC9 and the two uncharacterized proteins was ∼35%, their lengths were totally different (Fig. S5). This indicates that AcrIC9 from *R. capsulatus* is unique to this species. Since it is difficult to generalize the di­sulfide bond-mediated dimerization of AcrIC9, it is still possible that dimer formation is an artefact due to the experimental setup.

The inhibitory mechanism of many small-sized highly acidic Acrs (*e.g.* AcrIF14 and AcrIC5) is to bind to the target DNA-binding site in the CRISPR–Cas system (Liu *et al.*, 2019 ▸; Rollins *et al.*, 2019 ▸; Kang & Park, 2022 ▸). In addition, the most common inhibitory mechanism by Acrs is to inhibit target DNA recognition by the cascade by direct interaction with the latter’s component proteins (*e.g.* AcrIF1, AcrIF2, AcrIF4, AcrIF6, AcrIF7, AcrIF8, AcrIF9, AcrIF10 and AcrIF14) (Gabel *et al.*, 2021 ▸; Zhang *et al.*, 2020 ▸; Ka *et al.*, 2020 ▸). Because AcrIC9 is also a small-sized highly acidic protein, we speculate that it might use the first one. To provide evidence for this assumption, we found that AcrIC9 directly binds to Cas7 in the type IC CRISPR–Cas from *N. lactamica*. Interestingly, the dimeric form of AcrIC9 did not bind to Cas7, indicating that only the monomer can inhibit the type IC CRISPR–Cas system. Since the di­sulfide bond-mediated dimer formation of AcrIC9 blocks the binding capability of AcrIC9 to the cascade, this process could be important for Acr activity regulation, which is something to be determined in the future. Indeed, potential Acr activity regulation by di­sulfide bond formation has recently been proposed with the case of AcrIIC1 (Zhao *et al.*, 2022 ▸). However, because AcrIIC1 dimer was formed by two di­sulfide bonds with two conserved cysteines, this might not be the same case with our AcrIC9 system that is mediated by only a single di­sulfide bond with unconserved cystein.

The structure and surface features of AcrIC9 are similar to the ε subunit of RNA polymerase, which is known to control access to DNA to proceed the RNA polymerase reaction (Newing *et al.*, 2020 ▸; Keller *et al.*, 2014 ▸). Therefore, AcrIC9 could work similarly by occupying the target DNA-binding site for Cas7 in type IC CRISPR–Cas, thereby mimicking the *in situ* DNA process [Fig. 6 ▸(*d*)]. Our structural and biochemical characterization of AcrIC9 will help to uncover the molecular details of the inhibitory process of AcrIC9 against the type IC CRISPR–Cas system in the near future.

## Supplementary Material

Supporting information. DOI: 10.1107/S2052252523007236/lz5064sup1.pdf

PDB reference: AcrIC9, 8hjj

## Figures and Tables

**Table 1:** Structural data and refinement statistics for AcrIC9 Values for the outermost resolution shell are in parentheses.

Structural data	
Space group	*P*3_1_12
Unit-cell parameters	
*a*, *b*, *c* (Å)	69.41, 69.41, 89.04
*α, β, γ* (°)	90, 90, 120
Resolution range (Å)	28.4–2.4
Total reflections	195725
Unique reflections	9785
Multiplicity	20.0
Completeness (%)	99.74 (100)
Mean *I*/σ(*I*)	15.03 (2.23)
*R*_merge_ (%)†	29.93 (20.95)
*R*_meas_ (%)	29.68
*R*_p.i.m._	0.066
Wilson *B* factor (Å^2^)	43.32
CC_1/2_	0.999 (0.691)
Refinement	
Resolution range (Å)	28.4–2.4
Reflections	9768
*R*_work_ (%)	21.44
*R*_free_ (%)	26.92
No. of molecules in the ASU	3
No. of non-hydrogen atoms	1850
Macromolecules	1764
Solvent	86
Average *B*-factor values (Å^2^)	45.28
Macromolecules	46.68
Solvent	42.45
Ramachandran plot	
Favored/allowed/outliers (%)	100.0/0.0/0.0
Rotamer outliers (%)	1.14
Clashscore	12.09
RMSD bonds (Å)/angles (°)	0.008/1.04

† *R*_merge_ = Σ_*h*_Σ_*i*_|*I*(*h*)_*i*_ − 〈*I*(*h*)〉|/Σ*_h_* Σ*_i_* *I*(*h*)*_i_*, where *I*(*h*) is the observed intensity of reflection *h* and 〈*I*(*h*)〉 is the average intensity obtained from multiple measurements.
