# Supplementary material for: The structure of AcrIC9 revealing the putative inhibitory mechanism of AcrIC9 against the type IC CRISPR–Cas system
Source: IUCrJ. 2023 Sep 1;10(Pt 5):624–34. doi: 10.1107/S2052252523007236 (PMC10478522; doi:10.1107/S2052252523007236)
Supplement: Supplementary file 1 [file m-10-00624-sup1.pdf]

IUCrJ

Volume 10 (2023)

Supporting information for article:

The structure of AcrIC9 revealing the putative inhibitory mechanism of AcrIC9 against the type IC CRISPR–Cas system

Yong Jun Kang, Ju Hyeong Kim, Gwan Hee Lee, Hyun Ji Ha, Young-Hoon Park, Eunmi Hong and Hyun Ho Park

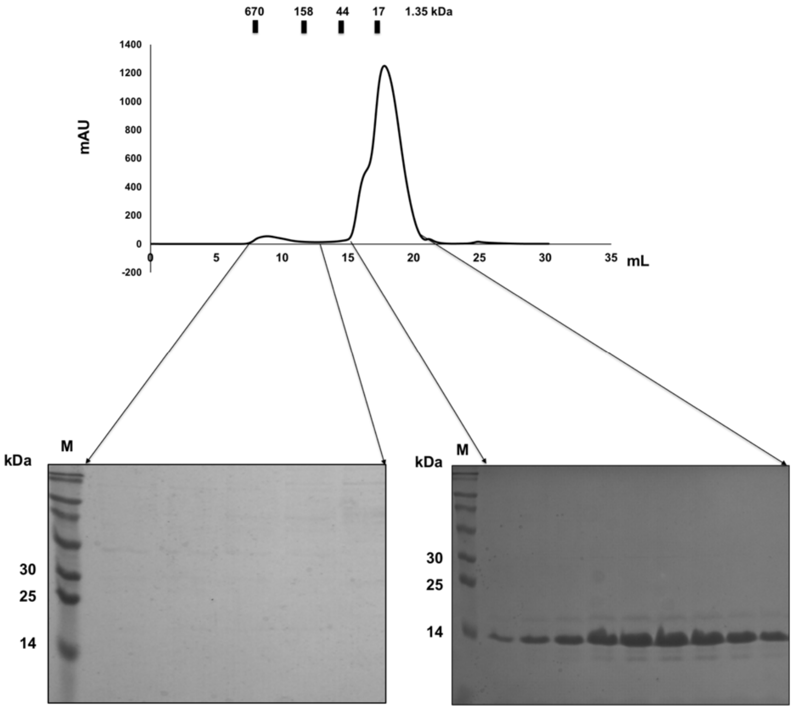

Figure S1 SEC profile analysis of AcrIC9. The SDS-PAGE gels produced by AcrIC9 are provided under the SEC profiles. Loaded fractions for SDS-PAGE were indicated by black arrows.

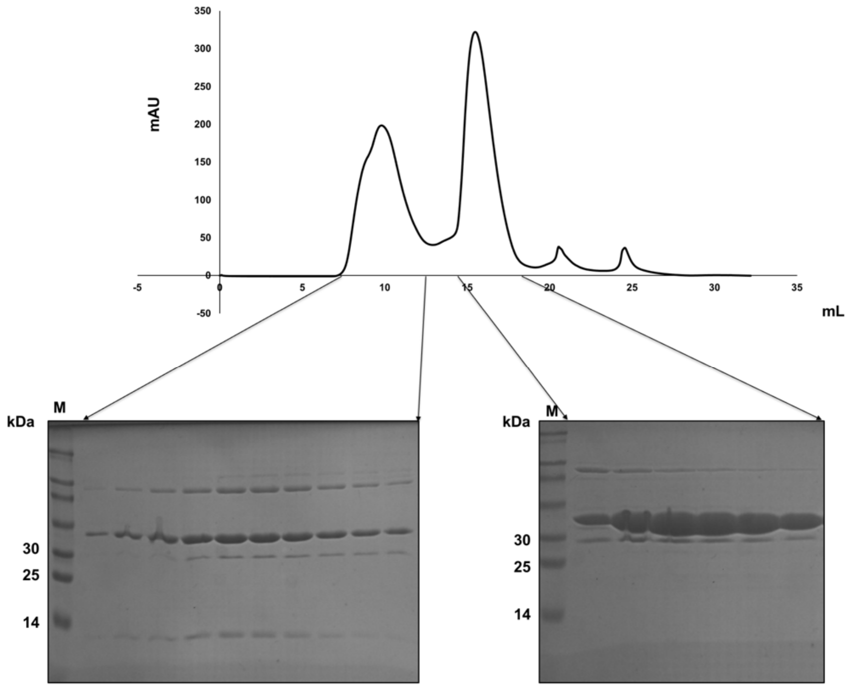

Figure S2 SEC profile analysis of the type I-C Cascade from *Neisseria lactamica*. The SDS-PAGE gels produced by the type I-C Cascade are provided under the SEC profiles. Loaded fractions for SDS-PAGE were indicated by black arrows.

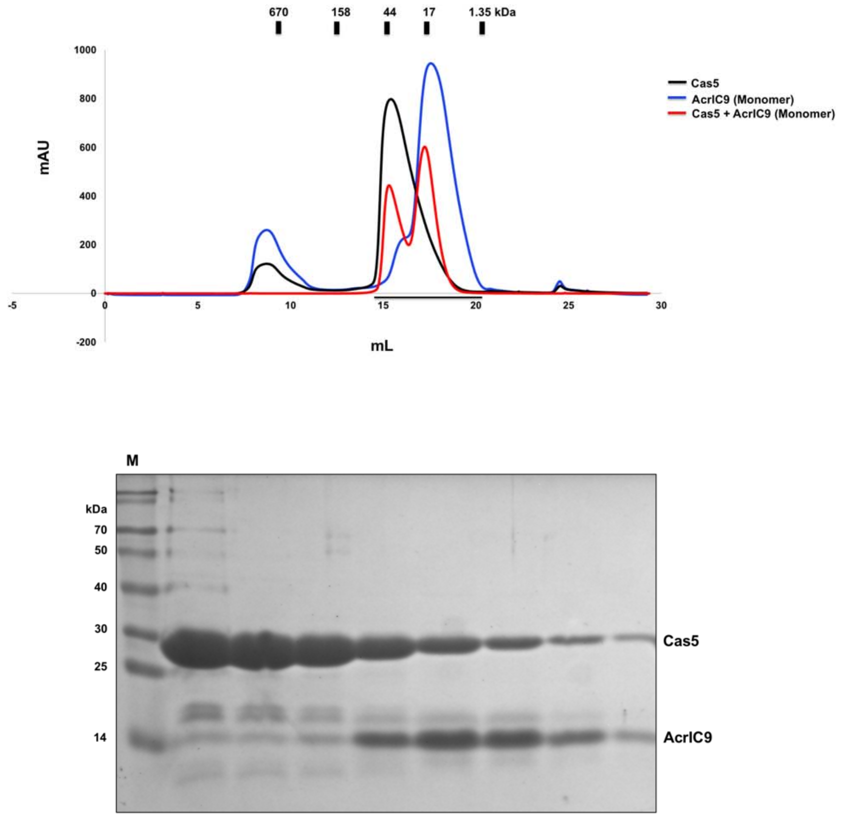

Figure S3 Interaction analysis between AcrIC9 and Cascade subunit Cas5 via SEC: the black, red, and blue lines indicate Cas5, a mixture of AcrIC9 and Cas5, and monomeric AcrIC9, respectively. The SDS-PAGE gels produced by the mixture of AcrIC9 and Cas5 are provided under the SEC profiles. Loaded fractions for SDS-PAGE were indicated by black and green bars.

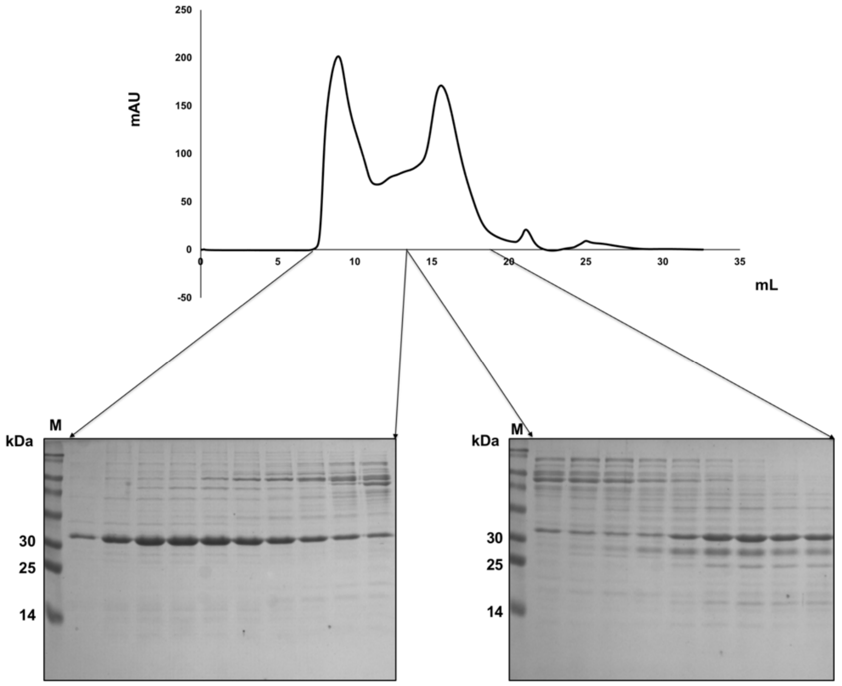

Figure S4 SEC profile analysis of Cas7. The SDS-PAGE gels produced by the Cas7 are provided under the SEC profiles. Loaded fractions for SDS-PAGE were indicated by black arrows.

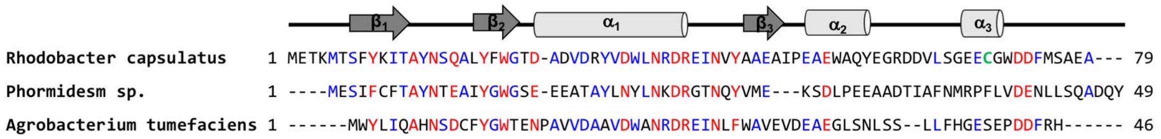

Figure S5 Sequence alignment of putative AcrIC9 homologs from different species. Mostly conserved and partially conserved residues are colored in red and blue, respectively.

Table S1 Summary of the binding affinity of NlaCascade/crRNA complex to rcAcrlC9

Parameters	
k_a (1/Ms)	$9.354x10^{4}$
k_d (1/s)	$4.042x10^{-3}$
K_D (M)	$4.321x10^{-8}$
